# *Agrocybe cylindracea* polysaccharides and polysaccharides-conditioned fecal microbiota transplantation similarly restore ciprofloxacin-induced microbial dysbiosis and improve intestinal barrier function: a comparative study

**DOI:** 10.3389/fimmu.2026.1841989

**Published:** 2026-07-13

**Authors:** Aamna Atta, Muhammad Naveed, Jinting Liu, Immad Ansari, Renzhen Ma, Xiyu Wang, Bin Feng

**Affiliations:** 1 College of Basic Medical Science, Dalian Medical University, Dalian, China; 2 Department of Traditional Chinese Medicine, Dalian University Affiliated Xinhua Hospital, Dalian, China

**Keywords:** *Agrocybe cylindracea*, polysaccharides, prebiotic, ciprofloxacin-induced dysbiosis, gut microbiota dysbiosis, intestinal barrier function, fecal microbiota transplantation

## Abstract

**Introduction:**

Mushroom consumption has been associated with various health benefits due to their recognized nutritional value. This study examines the ability of *Agrocybe cylindracea* polysaccharides (ACP) to reverse antibiotic-induced intestinal dysbiosis and evaluates their prebiotic potential in mitigating antibiotic-associated diarrhea.

**Methods:**

Male BALB/c mice aged 4–5 weeks were divided into five groups: normal control, ciprofloxacin (CIP)-treated, natural recovery, ACP treatment, and ACP-derived fecal microbiota transplantation (ACP-FMT). All intervention groups received CIP for 14 days, followed by their respective treatments for a further 14 days. Gut microbiota modifications were investigated using the Illumina MiSeq platform.

**Results:**

CIP administration markedly decreased bacterial diversity and richness, elevating pathogenic bacteria (*Proteobacteria, Enterococcus*, and *Bacteroides*) that persisted in the natural recovery group. Both ACP and ACP-FMT effectively counteracted dysbiosis, increasing beneficial genera including *Ruminococcaceae, Lachnospiraceae*_NK4A136, and *Firmicutes*. ACP and ACP-FMT restored mucin-2 biosynthesis and tight junction protein expression. ACP also reduced pro-inflammatory mediators including IL-6, IL-17, TNF-α, and IL-1β.

**Discussion:**

These findings highlight the prebiotic potential of ACP in restoring intestinal health, with therapeutic effects transferable via fecal microbiota transplantation, supporting their application as functional food ingredients for gut microbiota restoration.

## 1 Introduction

The human gastrointestinal tract harbors a highly complex and dynamic microbial ecosystem, primarily composed of bacteria and fungi, viruses, and archaea. This microbial consortium plays a fundamental role in supporting host health by influencing numerous physiological processes such as nutrient metabolism, immune maturation, intestinal barrier integrity, and pathogen defense through colonization resistance (1, 2). By generating metabolites like short-chain fatty acids (SCFA), the microbiota sustains epithelial health, modulates immune responses, and contributes to overall metabolic homeostasis (3–5). Disturbance of this delicate microbial equilibrium, commonly called dysbiosis, is now firmly associated with a variety of health disorders, including obesity, inflammatory bowel disease (IBD), type 2 diabetes, and even neurological conditions like depression and Parkinson’s disease (6–8). Antibiotic usage is particularly concerning among the many causes of dysbiosis due to its non-selective action against both pathogenic and beneficial microorganisms. Even brief exposure to broad-spectrum antibiotics can induce prolonged alterations in microbial diversity and metabolic activity (9, 10). The overuse and misuse of antibiotics, particularly fluoroquinolones such as ciprofloxacin (CIP), has emerged as a critical global public health concern, with fluoroquinolone-resistant *Escherichia coli* now recognized as one of the leading pathogen-drug combinations driving antimicrobial resistance-associated mortality worldwide (11). Beyond resistance, antibiotic-induced dysbiosis carries significant clinical consequences, including antibiotic-associated diarrhea, *Clostridioides difficile* infection, and prolonged disruption of immune and metabolic homeostasis (10, 12). Existing restorative approaches, including probiotic supplementation and fecal microbiota transplantation, are constrained by variable therapeutic outcomes and considerable regulatory and safety barriers, highlighting the pressing demand for safer, more reliable, and scalable strategies to rehabilitate gut microbial equilibrium following antibiotic-induced disruption (13, 14). CIP, a widely used fluoroquinolone antibiotic, is prescribed for the treatment of urinary, respiratory, and gastrointestinal infections. Its bactericidal effect stems from the inhibition of DNA gyrase and topoisomerase IV enzymes, which are important for replication of bacterial DNA (15). Despite its clinical efficacy, CIP is known to disturb the gut microbiota (16). It depletes commensal taxa such as *Firmicutes* and *Alistipes*, while favoring the overgrowth of *Proteobacteria*, notably pro-inflammatory species like *Escherichia coli* and *Enterobacter* spp (17–19). These shifts are commonly associated with decreased SCFA production, heightened mucosal permeability, and amplified immune responses 20). Crucially, spontaneous microbial recovery following antibiotic cessation is often incomplete or delayed. In some cases, the microbiota fails to convert to its pre-antibiotic configuration for a long period of time, if at all, depending on host genetics, diet, and environmental exposures (21). This persistent dysbiosis can increase susceptibility to chronic inflammatory conditions and recurrent infections, highlighting the need for therapeutic strategies that actively facilitate microbiota restoration and functional rehabilitation. Fecal microbiota transplantation (FMT) is currently the most effective clinical intervention for severe dysbiosis, especially in patients suffering from recurrent Clostridioides difficile infection (rCDI), where cure rates exceed 90% (12, 22). By introducing a full microbial group from a healthy donor, FMT boosts SCFA production, re-establishes ecological balance, and restores colonization resistance. Its application has expanded to include IBD, metabolic syndrome, and hepatic encephalopathy, where outcomes have been encouraging but inconsistent (23, 24). However, several limitations restrict the broader use of FMT. Therapeutic outcomes are highly donor-dependent, with only a minority of donors, so-called “super-donors”, consistently yielding favorable responses (25). Moreover, despite rigorous screening protocols, concerns remain regarding the inadvertent transmission of infectious agents, including antibiotic-resistant organisms (26). Regulatory difficulties further complicate its implementation, especially due to the complexities of standardizing donor material and ensuring batch-to-batch reproducibility (27). These restrictions have sparked interest in alternative microbiota-targeted approaches, such as the use of defined microbial groups and prebiotics. Prebiotics are non-digestible food components that particularly stimulate the growth and activity of beneficial microbes. Among novel prebiotic candidates, polysaccharides derived from edible and medicinal mushrooms have shown remarkable immunomodulatory and gut barrier-enhancing properties. *Agrocybe cylindracea*, an edible basidiomycete fungus, synthesizes bioactive polysaccharides (ACP) with demonstrated antioxidants, anti-inflammatory, and prebiotic activities (28). In our previous work, ACP was extracted, physiologically characterized, and shown to ameliorate DSS-induced colitis by improving the intestinal barrier and immune system through the gut–liver axis (29), providing the foundation for its therapeutic investigation in the present study. ACP has been reported to suppress the abundance of pro-inflammatory taxa, while favorably modulating gut microbiota composition by promoting beneficial genera such as *Lactobacillus* and *Bifidobacterium* (30). In experimental models of chemically induced colitis, ACP administration has been shown to attenuate inflammation by reducing pro-inflammatory cytokine levels, such as LPS, IL-6, and IL-1β, primarily via inhibition of the p38 MAPK signaling cascade (31). Additionally, ACP promotes mucosal healing by enhancing mucin secretion (MUC2) and upregulating tight junction proteins, including ZO-1 and Occludin, thereby improving epithelial barrier integrity (32). These multifaceted actions suggest that ACP could restore microbial and immune homeostasis through mechanisms comparable to those of FMT, while offering advantages in terms of safety, scalability, and standardization. Notably, microbiota derived from ACP-treated hosts may possess therapeutic properties, raising the possibility that such material could be employed in a modified form of microbiota transplantation (33, 34). Despite growing evidence for the prebiotic potential of mushroom-derived polysaccharides, how ACP specifically supports recovery from antibiotic-induced dysbiosis remains unclear. Here, we used CIP-treated mouse model to test ACP both as a direct therapeutic and as a microbiota-shaping agent, uniquely pairing it with FMT derived from ACP-treated donors to separate its direct effects from those mediated through the gut microbiota. We hypothesize that ACP will strengthen the intestinal barrier, reduce inflammation, and restore microbial balance, with its conditioned FMT mirroring these benefits, positioning ACP as a practical and mechanistically grounded alternative to conventional microbiota restoration.

## 2 Materials and methods

### 2.1 ACP extraction

Fruiting bodies of *Agrocybe cylindracea* were commercially sourced from Yunnan Congrong Economic and Trade Co., Ltd. (Kunming, China). The mushrooms were obtained from a commercial supplier that specializes in edible fungi. The supplier confirmed species identity using standard morphological authentication and quality control measures that align with established commercial practices for this well-known species. Before processing, the mushroom samples were thoroughly rinsed with distilled water to clean surface contaminants and water-soluble impurities. They were then dried with hot air at 60 °C until they reached a stable, constant mass. We ground the dried tissue mechanically and then screened it through a 40-mesh sieve (0.42 mm aperture) to obtain a fine, uniform powder for further processing. We used 100 g of the powdered material for two hot-water extraction cycles to get polysaccharides. The powder was mixed with distilled water, and with the first extraction cycle at a solid-to-liquid ratio of 1:30 (w/v), kept at 80 °C for 4 h, placed in a water bath with a thermostat. The resulting supernatant was centrifuged at 5,000 rpm for 10 min, and the pellet was retained for a second extraction step. The remaining material was mixed back into distilled water at a 1:20 (w/v) ratio and kept at the same temperature for 2 h. Then, it was centrifuged at 5,000 rpm for 15 min. The supernatants from both extraction cycles were combined and allowed to cool to room temperature. Deproteinization was carried out by introducing trichloroacetic acid (TCA) to the pooled extract at a final concentration of 1.5% (w/v). To allow full precipitation of proteins, the mixture was stored at 4 °C overnight. After that, 2 M NaOH was used to bring the pH of the solution back to 7.0, and the proteins that had formed were removed by spinning them at 5,000 rpm for 10 min at 4 °C. Using a rotary evaporator kept at 60 °C, the clarified supernatant slurry was concentrated to one-fifth of its original volume under reduced pressure. Then, in the next step, to prepare crude polysaccharides, four times the volume of absolute ethanol was added to the concentrated solution, and the mixture was stored at 4 °C overnight. The precipitation was separated by centrifugation at 5,000 rpm for 10 min at 4 °C. Then, the mixture was vacuum-dried at 30 °C to help evaporate remaining ethanol. The final product was freeze-dried at −20 °C, the crude polysaccharide fraction of *A. cylindracea*, called ACP (29).

### 2.2 Monosaccharide composition of ACP

High-performance liquid chromatography (HPLC) in conjunction with pre-column derivatization using 1-phenyl-3-methyl-5-pyrazolone (PMP) was used to evaluate the monosaccharide composition of ACP. This method was somehow modified from a previously published approach (35). For acid hydrolysis, 50 mg of ACP was exposed to 2 M trifluoroacetic acid (TFA) at 120 °C for six hours. After full hydrolysis, the remaining TFA was removed by repeatedly co-evaporating with methanol at lower pressure. After neutralizing the resultant hydrolysate to the proper pH, it was derivatized at 70 °C using PMP reagent. Before chromatographic analysis, the derivatized products were separated into chloroform, and the aqueous phase was cleared by passing it through a 0.22 µm nylon membrane filter (Millipore, Westborough, MA, USA). An Agilent 1260 Infinity II chromatographic system with a ZORBAX Eclipse XDB-C18 reversed-phase column (4.6 × 250 mm, 5 µm particle size; Agilent Technologies, Santa Clara, CA, USA) was used for HPLC separation. The mobile phase was supplied isocratically at a flow rate of 1.0 mL/min and consisted of 0.1 M phosphate buffer (pH 6.7) and acetonitrile at a volumetric ratio of 82:18 (v/v). The absorbance wavelength used for analyte detection was 254 nm. By comparing experimentally obtained retention times with authenticated PMP-derivatized reference standards obtained from Sigma-Aldrich (St. Louis, MO, USA), such as glucose (Cat. No. G8270), galactose (Cat. No. G5388), mannose (Cat. No. M2069), and glucuronic acid (Cat. No. G5269), monosaccharide identification was accomplished.

### 2.3 Animals and experimental design

**Figure 1:**
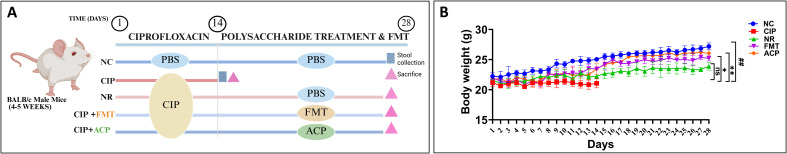
Experimental protocol and body weight changes in ciprofloxacin (CIP)-treated mice following ACP and ACP-FMT intervention. **(A)** Animal plan of the study enabling assessment of *A. cylindracea* polysaccharide group (ACP) and the effects of ACP-FMT on antibiotic-induced mice. The schematic illustrates the protocol for the study, which included a range of experimental and control groups across different phases. The timeline emphasizes the adaptation phase, model development using 50 mg kg^-^¹ of CIP for 14 days, and the treatment 300 mg kg^-^¹ ACP, and feces 160 mg mL^-^¹ of PBS, 200 µL of oral gavage for the ACP-FMT treatment group for the next 14 days. The group includes PBS as the normal control group, CIP as the model group, NR (natural recovery) group, the dosage of ACP, and ACP-FMT as treatment groups. **(B)** Effect of polysaccharide treatment on body weight. Comparison between ACP, ACP-FMT, and NR group is denoted by (ns: *p < 0.05: **p < 0.01) compared to CIP, while the comparison between the CIP and the NC group is denoted by (^##^*p* < 0.01).

Forty BALB/c male mice, aged 4 to 5 weeks, were obtained from the SPF facility at Dalian Medical University to create the CIP-induced antibiotic model. Animal studies were carried out according to institutional policies and ethical standards (Registration No. AEE24192). Under a conventional laboratory environment (temperature: 22 °C ± 2 °C, humidity: 50% ± 5%, light-dark cycle: 12 h), a week of adaptation began with free access to standard food and water. The mice were divided into five experimental groups at random, after the acclimatization phase (n=8 per group). The groups consisted of: (1) normal control (NC) group, which received no treatment; (2) ciprofloxacin (CIP) model group; (3) natural recovery (NR) group; (4) fecal microbiota transplantation (FMT) group; and (5) *A. cylindracea* polysaccharides (ACP) treatment group, as depicted in **Figure 1A**. Except for the NC group, all other groups administered 50 mg kg^-^¹ of CIP via oral gavage once daily for 14 consecutive days. This dosage has been shown in previous mouse studies to reliably induce gut microbiota dysbiosis while remaining well tolerated (36, 37). On day 14 of the experiment, fresh fecal samples were collected from the CIP group under aseptic conditions, after which these animals were humanely euthanized using CO_2_ asphyxiation. The NR group, as a vehicle control, was given an equivalent volume of PBS, whereas ACP group received a daily oral gavage dose of 300 mg kg^-^¹ *A. cylindracea* polysaccharides solution. A thorough pilot dose-ranging study that assessed four concentrations (100, 150, 300, and 400 mg kg^-^¹) used as the basis for the 300 mg kg^-^¹ dose of ACP. The findings clarified that lower doses (100 mg kg^-^¹) exhibited little therapeutic value, levels between 150–300 mg kg^-^¹ showed notable biological effects. Interestingly, compared to the 300 mg kg^-^¹ dose, the highest tested dose (400 mg kg^-^¹) did not show more beneficial results, indicating a plateau in the dose-response relationship. Because of its balanced efficacy and safety effects, 300 mg kg^-^¹ was chosen as the ideal dose for further study (29). For the FMT procedure, fresh fecal samples were collected daily from donor mice in ACP group throughout the concurrent 14-day ACP treatment period (days 15–28), during which ACP administration was actively reshaping the donor gut microbiota toward a restored microbial composition, and a 200 µL oral gavage dose was used. These samples were homogenized in ice-cold PBS at a ratio of 160 mg fecal matter to 1 mL of buffer as soon as possible. The combined feces were subsequently filtered through a 70 µL filter mesh to exclude contaminants. To remove particle debris, the resultant mixture was mixed and centrifuged at 4000 rpm for 15 min at 4 °C. Together, these processing steps were intended to yield a microbiota-enriched supernatant with minimal carry-over of any residual dietary polysaccharides, including ACP, from the donor fecal material. Before being transplanted, the supernatant was meticulously put together and divided into sterile cryovials for refrigeration at 4 °C (38, 39). This FMT protocol was designed to investigate whether the observed therapeutic effects of ACP in a previous study could be attributed to its modulation of gut microbiota composition and function. The experimental timeline concluded on day 29, at which point all remaining mice were euthanized using approved methods after feces collection. The entire colon was aseptically removed from each mouse, with the distal colon segment immediately fixed in 4% neutral-buffered formalin for histological examination. All tissue samples and serum were stored at −80 °C until further molecular examination to ensure preservation of biological integrity. This comprehensive experimental design allowed for the thorough assessment of the therapeutic potential of ACP in the antibiotic-induced dysbiosis model and its mechanism of action via multiple analytical approaches.

### 2.4 Body weight assessment

The daily weight of every mouse within each group was documented throughout the duration of the experiment.

### 2.5 Histological analysis of colon tissues

To guarantee the best possible preservation of tissue architecture, the distal colon segments were meticulously dissected and immediately fixed in freshly made 4% paraformaldehyde (pH 7.4) for 24 hours at 4 °C. Following fixation, the samples were cleaned in xylene, embedded in paraffin wax, and dehydrated using a graded ethanol series (70%, 80%, 95%, and 100%). Pieces with 3-µm-thick were cut with a rotary microtome and placed on glass slides to be stained. The sections were first deparaffinized in xylene for ten min and then rehydrated using a descending ethanol gradient (100%, 95%, 70%, and 50%; five min each) for H&E (Solarbio, Cat.No.G1120) staining, in accordance with the protocol established (40), which is essential for optimal nuclei staining. Mayer hematoxylin was used to stain nuclei for three min, and excess dye was removed by rinsing them under running water for five min. Eosin Y was used for cytoplasmic staining (2 min). The sections were covered with mounting solution (Solarbio, cat-G8590), cleaned in xylene, and dehydrated using increasing ethanol concentrations (95% and 100%). Slide-mounted tissue sections were subjected to a blinded histological evaluation under a microscope (Leica Microsystems, Wetzlar, Germany). A consistent scoring system was used to quantify parameters like tissue morphology, inflammation, and regeneration (**Supplementary Table 1**).

### 2.6 IHC for Mucin-2 detection

Tissue samples fixed in paraffin were put onto positively charged slides to examine the effect of ACP on Mucin-2 expression after antibiotic treatment. These slides were incubated at 65 °C for 2 h. The slides were then rehydrated using a sequence of decreasing ethanol concentrations after being deparaffinized twice for 10 min using xylene. The slides were subsequently treated with citrate buffer using microwave heating to aid in antigen retrieval. The tissue slices were treated with H_2_O_2_ for 20 min after the antigens were extracted to prevent endogenous peroxidase activity. The immunohistochemistry (IHC), staining kit (Biotechnologies Biotechnology, Beijing, China) protocol was followed for all subsequent steps. The tissue slices were incubated at 4 °C for an entire night, after preparing and applying a primary antibody against mucin-2 (Proteintech, 27675-1-AP; 1:1,000). The antibody incubation was finished, and the slides underwent three 10 min PBS washes to protect it from any unbound antibody. After that, a species-related secondary antibody was used and left to incubate for 1 h at room temperature. After further PBS washes, 3,3′-diaminobenzidine (DAB) substrate was applied for five min to perform chromogenic development. The slides were then washed under running tap water for 10 min to stop the reaction and extra chromogen binding. To reduce background staining and improve nuclear contrast, counterstaining was performed with hematoxylin for 5 min, followed by a 10 min wash in slow-running tap water. To achieve optical transparency, the stained sections were cleaned in xylene after being dehydrated using a graded ethanol series. Neutral balsam mounting solution (Cat. No. G8590, Solarbio, Beijing, China) was used to apply coverslips. All stained slides were inspected under a light microscope, and immunolabeled cells were examined blindly to reduce observational bias. Deparaffinized and rehydrated sections were oxidized with 0.5% periodic acid for five min at room temperature to reveal carbohydrate moieties for periodic acid–Schiff (PAS) staining. The sections were thoroughly cleaned in distilled water for 10 min, then they were treated with Schiff reagent for 10 min to see goblet cells that contained mucin, and finally they were rinsed with tap water for eight min. Nuclei were highlighted with a 7 min hematoxylin counterstain. The parts were then mounted after being dried and cleansed in xylene. A light microscope (Leica Microsystems, Wetzlar, Germany) was used to blindly collect digital images of stained sections at magnifications of 10× and 20×. ImageJ software was then used for quantitative analysis. Mucosal thickness was measured from the muscularis mucosae to the luminal surface, and goblet cells were recognized by their PAS-positive mucin concentration. For statistical comparisons, each specimen’s mean values were utilized.

### 2.7 Tight junction proteins immunofluorescence

The expression of tight junction proteins in mouse colonic tissue was evaluated by immunofluorescence (IF) staining performed on formalin-fixed, paraffin-embedded tissue sections. Slides were first deparaffinized in xylene for 10 min, then progressively rehydrated through a graded ethanol to distilled water. Antigen retrieval was accomplished by heating the sections in citrate buffer by using a microwave oven under standardized conditions. Following retrieval, slides were washed three times with PBS, and endogenous peroxidase activity was quenched by treatment with hydrogen peroxide (H_2_O_2_) for 15 min, after which an additional PBS wash was performed to remove residual H_2_O_2_. Tissue sections were subsequently incubated overnight at 4 °C with the following primary antibodies: anti-ZO-1 (dilution 1:1,000; Cat. No. AF5145, Affinity Biosciences), anti-Occludin (dilution 1:400; Cat. No. 66378-1-Ig, Proteintech, Wuhan, China), and anti-Claudin-1 (dilution 1:1,000; Cat. No. 13050-1-AP, Proteintech, Wuhan, China). Following the incubation of the primary antibody, slides were rinsed with PBS and then incubated for one hour at room temperature with a goat anti-rabbit secondary antibody conjugated with fluorescein isothiocyanate (FITC) (Proteintech, Wuhan, China). Slides were rinsed three times with PBS for five min each after the secondary antibody was incubated. To make cell nuclei easier to see, nuclear counterstaining was carried out by adding, 4′,6-diamidino-2-phenylindole (DAPI) for 5 min. To remove observational bias, coverslips were placed on top of the stained sections, and each slide was blindly inspected under a fluorescence microscope. ImageJ was used to quantify the fluorescence signal intensity.

### 2.8 Serum preparation

Mice were sacrificed on the last day of the experimental endpoint, and the entire blood was promptly obtained through retro-orbital hemorrhage. Firstly, the blood was placed at room temperature for 2 h, and then centrifuged at 3,000 rpm for 20 min at room temperature to separate the serum. The resultant supernatant was then carefully aspirated and aliquoted. Before additional biochemical analysis, all serum samples were kept at −80 °C (41).

### 2.9 ELISA of serum inflammatory mediators

Serum concentrations of pro-inflammatory mediators, including IL-6 (Cat. No. MB-6368A), IL-17 (Cat. No. MB-2912A), TNF-α (Cat. No. MB-2868A), IL-1β (Cat. No. MB-2776A), and anti-inflammatory, IL-10 (Cat. No. MB-2912A), were quantified using commercially available mouse-specific enzyme-linked immunosorbent assay (ELISA) kits (Jiangsu Meibiao Biotechnology Co., Ltd., Jiangsu, China), in strict accordance with the manufacturer’s recommended protocol. Before initiating the assay, all kit reagents were placed at room temperature for 30 min. Aliquots of 50 µL from each serially diluted standard reference solution and test serum sample were dispensed into the designated microplate wells, followed by the addition of 50 µL of horseradish peroxidase (HRP)-conjugated detection reagent to each well. To allow immune complex formation, the plates were then incubated at 37 °C for 60 min. Following incubation, unbound reagents were removed by washing each plate five times with the provided washing buffer. By the addition of 50 µL of chromogen solutions A and B to all wells, the colorimetric detection reaction was initiated. Plates were incubated at 37 °C for an additional 15 min in the dark to allow color development. The enzymatic reaction was stopped by the addition of 50 µL of stop solution per well. Optical absorbance was measured at 450 nm within 15 min of stop solution addition using a microplate spectrophotometer. Cytokine concentrations in each sample were extrapolated from their respective standard curves generated using the kit-supplied reference standards.

### 2.10 DNA extraction from stool samples and 16s rRNA gene amplification for Illumina MiSeq sequencing

The PowerMax Stool DNA Isolation Kit (MoBio Laboratories, Carlsbad, CA, USA) was used in accordance with the manufacturer’s suggested protocol to extract total genomic DNA from mouse fecal samples. A NanoDrop spectrophotometer (Thermo Fisher Scientific, Waltham, MA, USA) was used to measure the concentration of extracted DNA, and electrophoretic separation on a 1% agarose gel was used to verify DNA integrity. The hypervariable V3–V4 region of the bacterial 16S rRNA gene was amplified by PCR, using the forward primer 338F (5′-ACTCCTACGGGAGGCAGCAG-3′) and the reverse primer 806R (5′-GGACTACHVGGGTWTCTAAT-3′). The thermocycling schedule included a first denaturation phase at 98 °C for 1 min, followed by 30 amplification cycles at 98 °C for 10 seconds, with primer annealing at 50 °C for 30 seconds, strand extension at 72 °C for 60 seconds, and a final extension step at 72 °C for 5 min. The first sample cohort was sequenced at Shanghai Yuanxin Biomedical Technology Co., Ltd. (Origin-gene, Shanghai, China), using the Illumina MiSeq platform (Illumina, San Diego, CA, USA). The QIIME pipeline (v1.9.0) was used for bioinformatic analysis and community inspection. Alpha diversity indices, such as the Shannon diversity index and observed species richness, were used to evaluate intra-sample microbial diversity. Beta diversity analyses utilizing weighted UniFrac distance-based principal coordinate analysis (PCoA) and non-metric multidimensional scaling (NMDS) were used to assess inter-sample compositional dissimilarity. Linear discriminant analysis effect size (LEfSe, v1.0), with an LDA score threshold of ≥ 2.0 and a significance level of *p* < 0.05, was used to identify microbial species that were differentially abundant across experimental groups. The Kyoto Encyclopedia of Genes and Genomes (KEGG) pathway database was used to perform functional annotation of the bacterial communities to deduce potential metabolic functions and ecological roles linked with the identified microbial assemblages. Detailed sequencing quality control parameters and bioinformatic pipeline information are available through the deposited raw data in the NCBI Sequence Read Archive (SRA) under BioProject accession number PRJNA1477999.

### 2.11 Data analysis

All statistical analyses were performed using GraphPad Prism software (version 10.2.3). Quantitative data are presented as mean ± standard deviation (SD) with a group size of five animals per experimental condition (*n* ≥ 3). Between-group differences were evaluated using ANOVA with Tukey’s *post hoc* test for multiple comparisons, statistical significance defined at a threshold of *p* < 0.05.

## 3 Results

### 3.1 Chemical characterization of *Agrocybe cylindracea* polysaccharides

**Figure 2:**
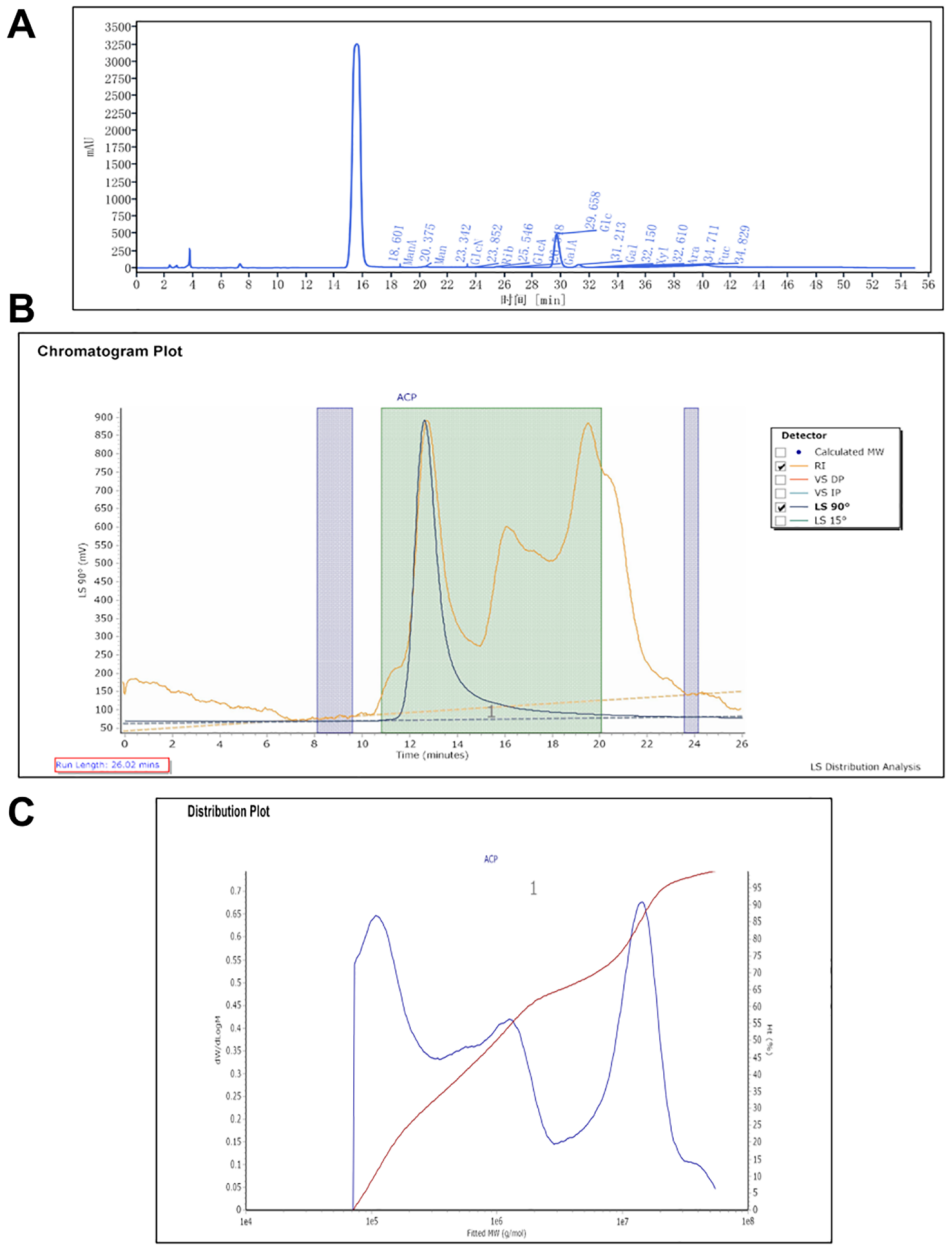
Physicochemical characterization of ACP: Monosaccharide Composition, Molecular Weight, and GPC Profile. **(A)** Characterization of *Agrocybe cylindracea* polysaccharides (ACP) by High-performance liquid chromatography (HPLC). ACP consists predominantly of glucose and galactose, accompanied by minor quantities of glucuronic acid and mannose. Additional trace components identified included ribose, galacturonic acid, mannuronic acid, fucose, arabinose, and xylose. Adapted with permission from Atta et al. (29) licensed under CC BY 4.0, doi: 10.3390/ijms26146805. **(B)** GPC chromatogram with refractive index (RI) detection showing elution profile over time. The cumulative distribution (red line) indicates polydisperse molecular weight characteristics. **(C)** Molecular weight distribution of ACP obtained through gel permeation chromatography. Molecular weight distribution plot showing bimodal distribution with major peaks approximately at 10^5^ and 10^7^ g mol^-1^.

HPLC investigation of the monosaccharide composition of ACP revealed that glucose was the predominant constituent, accounting for 87.13% of the total sugar content, followed by galactose (5.91%), glucuronic acid (2.72%), mannose (1.56%), ribose (1.01%), galacturonic acid (0.85%), mannuronic acid (0.48%), and trace amounts of fucose (0.27%), arabinose (0.04%), and xylose (0.02%) (**Figure 2A**; **Table 1**) (29). Furthermore, gel permeation chromatography analysis indicated that the crude ACP contained a substantial proportion of high-molecular-weight polysaccharides. This heterogeneous monosaccharide composition reveals the presence of structurally complex polysaccharides within ACP, which may be responsible for its biological actions. Forming the backbone of the complex bioactive polysaccharides, the dominant glucose content strongly suggests that glucan-type polysaccharides represent the primary structural components of ACP (**Figures 2B, C**).

**Table 1:** Monosaccharide composition of *Agrocybe cylindracea* crude polysaccharides.

Components	Concentration (mg/kg)	Percentage (%)
Mannuronic acid	1521.43	0.48
Mannose	4964.29	1.56
Ribose	3221.43	1.01
Glucuronic acid	8650	2.72
Galacturonic acid	2707.14	0.85
Glucose	276950	87.13
Galactose	18782.14	5.91
Xylose	75	0.02
Arabinose	128.57	0.04
Fucose	850	0.27

Adapted with permission from Atta et al. (29) licensed under CC BY 4.0, doi: 10.3390/ijms26146805.

### 3.2 Effect of ACP and FMT-ACP on body weight changes

Body weight was monitored daily throughout the 28-day experimental period. Following 14 days of CIP administration, the CIP-treated group (CIP) exhibited a significant reduction in body weight compared to the NC (21.04 ± 0.24; *p* < 0.01). During the subsequent 14-day treatment phase, the NR group showed gradual but incomplete weight restoration (22.59 ± 0.84 vs. CIP). In contrast, ACP treatment group and the ACP-FMT treated group showed significantly improved weight recovery, FMT (23.48 ± 1.53; *p* < 0.05), and ACP (23.84 ± 1.84; *p* < 0.01) compared to the CIP group. ACP and ACP-FMT therapeutic interventions revealed superior efficacy in restoring body weight compared to NR alone (**Figure 1B**).

### 3.3 ACP and FMT ameliorate histopathological alterations

**Figure 3:**
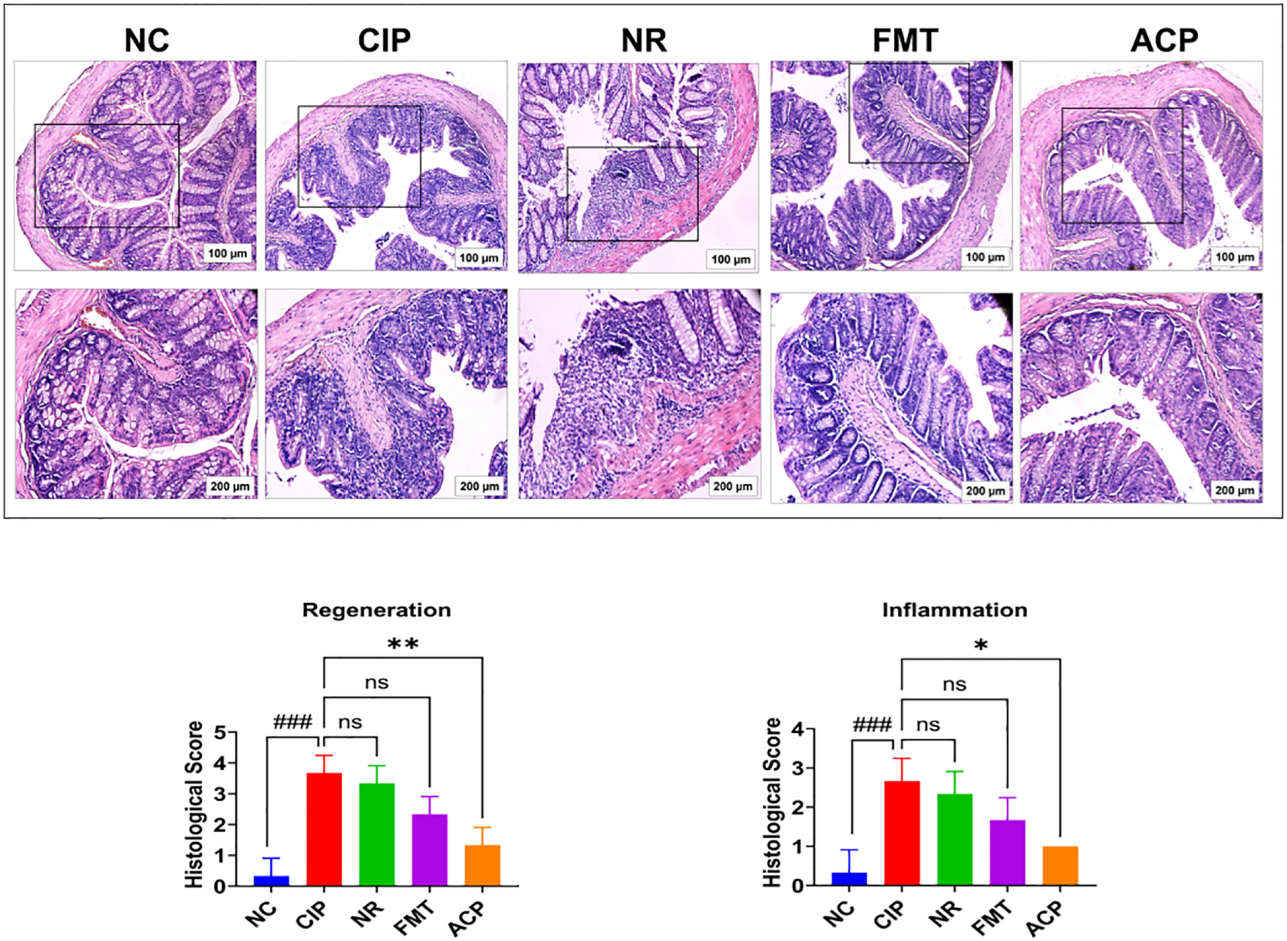
ACP and ACP-FMT ameliorate antibiotic-induced colonic histopathological damage. Histological evaluation of colon tissue after antibiotic treatment with mushroom polysaccharides (ACP) and ACP-FMT group in antibiotic-induced inflammation. H&E staining reveals histological changes in colon tissue among various experimental groups. Magnification: upper: 10×, lower: 20×; scale bars: 100 μm, 200 μm. Data are presented as mean ± SD, ^###^*p <* 0.001 CIP vs. NC; *p < 0.05, and **p < 0.01, NR, ACP, and ACP-FMT vs. CIP group.

Using hematoxylin and eosin (H&E) staining, morphological changes in colonic tissue across all five experimental groups were assessed by histological examination. Sections from the normal control group showed well-preserved colonic architecture, characterized by elongated, intact villi, clear crypts, and compact, organized tissue layers, consistent with healthy mucosal morphology. In contrast, mice subjected to 14 days of CIP treatment exhibited marked histopathological deterioration, manifested as villous shortening, structural irregularity, and disruption of epithelial barrier integrity. The pronounced infiltration of inflammatory cells into the lamina propria, a notable reduction in goblet cell density, and widening of the intercellular spaces between the mucosal and submucosal layers. These findings are aligned with antibiotic-induced colonic mucosal injury and dysbiosis-associated inflammation. The NR group demonstrated partial histological improvement relative to the CIP group; however, considerable pathological alterations persisted, and tissue architecture remained substantially different from that observed in the normal control. In comparison, both ACP-treated and ACP-FMT treated groups showed significant reversal of CIP-induced histopathological changes, including reduction in inflammatory cell infiltration and a noticeable restoration of goblet cells. Overall, the therapeutic intervention groups demonstrated substantially superior histological recovery compared to both the CIP and NR groups, suggesting a protective and restorative effect of ACP and ACP-FMT on antibiotic-compromised colonic mucosa (**Figure 3**).

### 3.4 ACP and FMT enhance mucin production and goblet cell function

**Figure 4:**
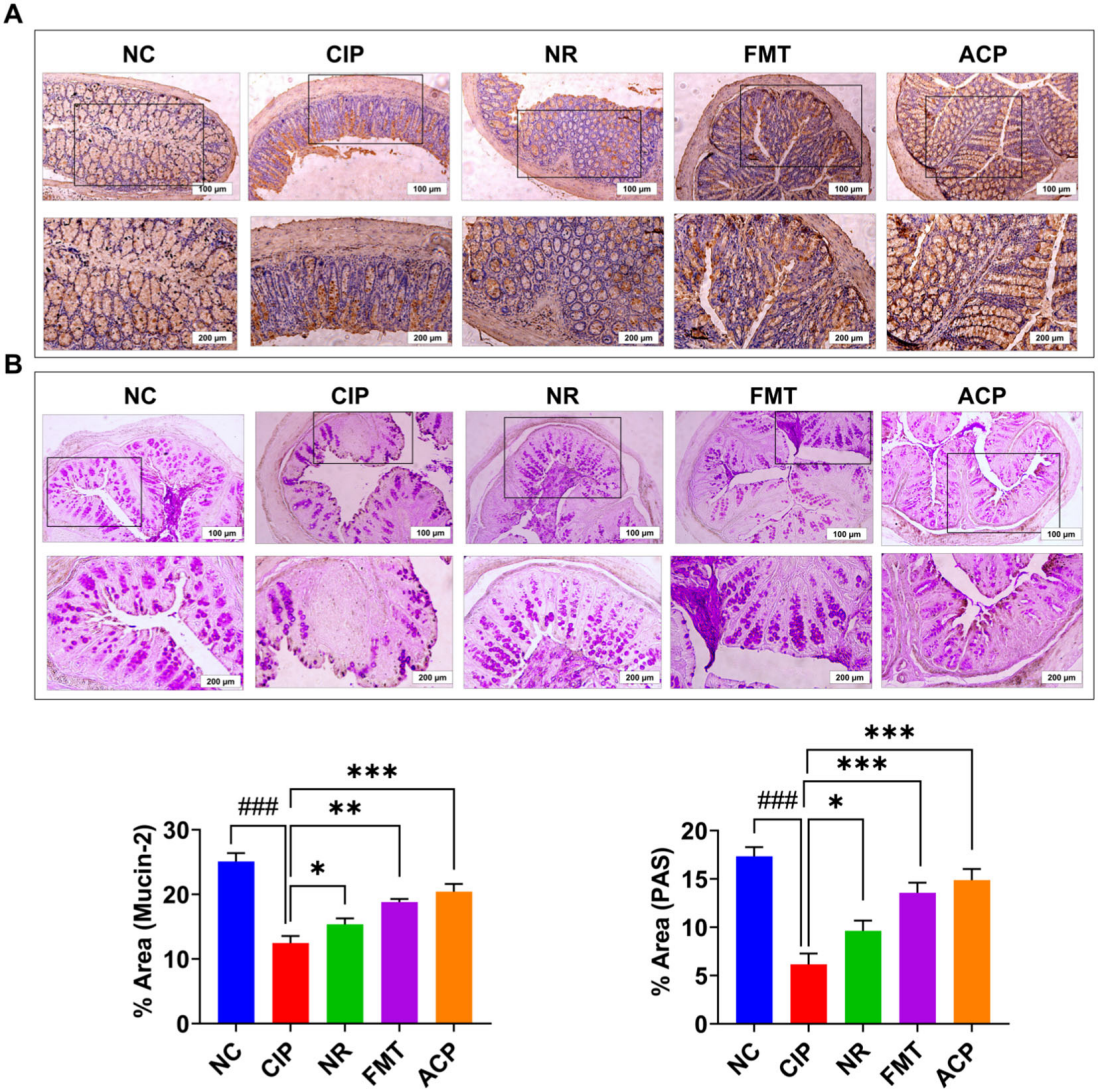
ACP and its ACP-FMT improve Mucin expression and goblet cell numbers in colon tissue. **(A)** Immunohistochemistry analysis for Mucin-2, revealing mucin expression in mice colon from different experimental groups; **(B)** PAS staining for goblet cells analysis. Magnification: (upper: 10×, lower: 20×; scale bars: 100 μm, 200 μm). Data are presented as mean ± SD, ^###^*p* < 0.001 CIP vs. NC; *p < 0.05, **p < 0.01, and ***p < 0.001 NR, ACP, and ACP-FMT vs. CIP group.

To analyze Mucin-2 expression in colon tissue across all experimental groups, Immunohistochemical analysis (IHC) was conducted. The CIP model group exhibited reduced mucus layer thickness, significantly decreased Mucin-2 expression, and increased the presence inflammatory cells as compared to NC. The NR group showed partial restoration but remained significantly lower than the NC. Both ACP and ACP-FMT treatment groups demonstrated significant progress in Mucin-2 production compared to the NR and CIP groups, with expression levels approaching normal control, as illustrated in **Figure 4A**. Periodic acid-Schiff (PAS) staining was implemented to quantify goblet cells across all groups. The CIP group exhibited reduced glycoprotein content and decreased goblet cell numbers, compared to NC. The NR group showed incomplete restoration of goblet cell populations. ACP and ACP-FMT treatments effectively restored and increased goblet cell populations and mucin expression, showing significantly higher counts than the NR and CIP groups, as shown in **Figure 4B**.

### 3.5 ACP and FMT restore tight junction protein expression

**Figure 5:**
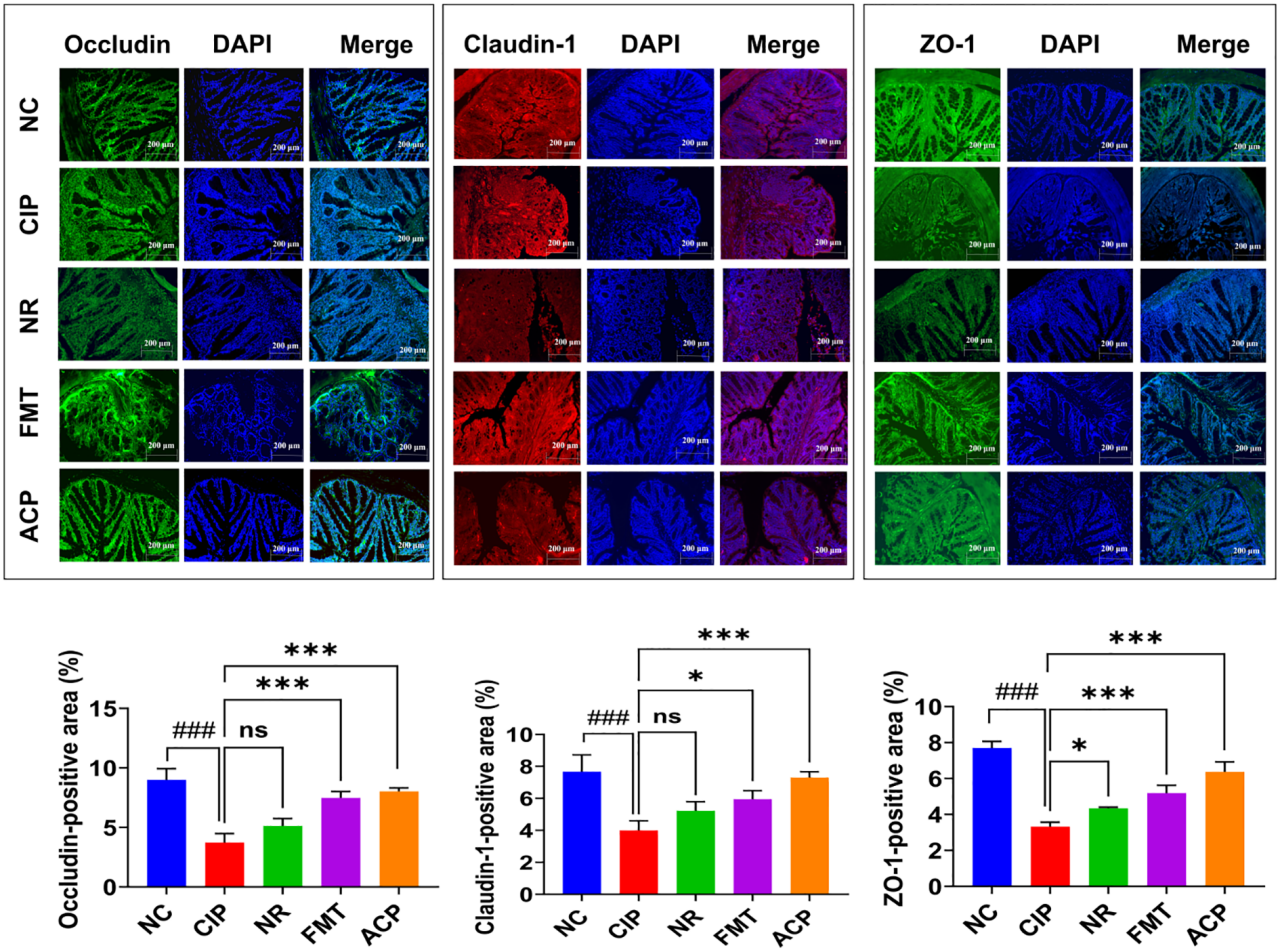
ACP and ACP-FMT recover, colonic tight junction proteins expression. Immunofluorescent staining of tight junction proteins Occludin, Claudin, and ZO-1 in colon tissue. Images were captured at 20× magnification (scale bar: 200 μm), and quantification graphs for each marker were provided. Data are presented as mean ± SD, ^###^*p* < 0.001, CIP vs. NC; *p < 0.01 and ***p < 0.001, ACP and ACP-FMT vs. CIP group.

To assess tight junction protein expressions (ZO-1, Claudin-1, and Occludin) in colon tissue of all experimental groups, Immunofluorescence (IF) analysis was conducted. Tight junction protein expression was significantly decreased in the CIP group compared to the normal control. The NR group exhibited partial restoration but remained significantly lower than the normal control. Both ACP and ACP-FMT treatments significantly upregulated the expression of all three tight junction proteins compared to both the CIP and NR groups, indicating superior restoration of intestinal barrier function through therapeutic intervention rather than NR alone (**Figure 5**).

### 3.6 ACP and FMT reduce pro-inflammatory cytokine levels

**Figure 6:**
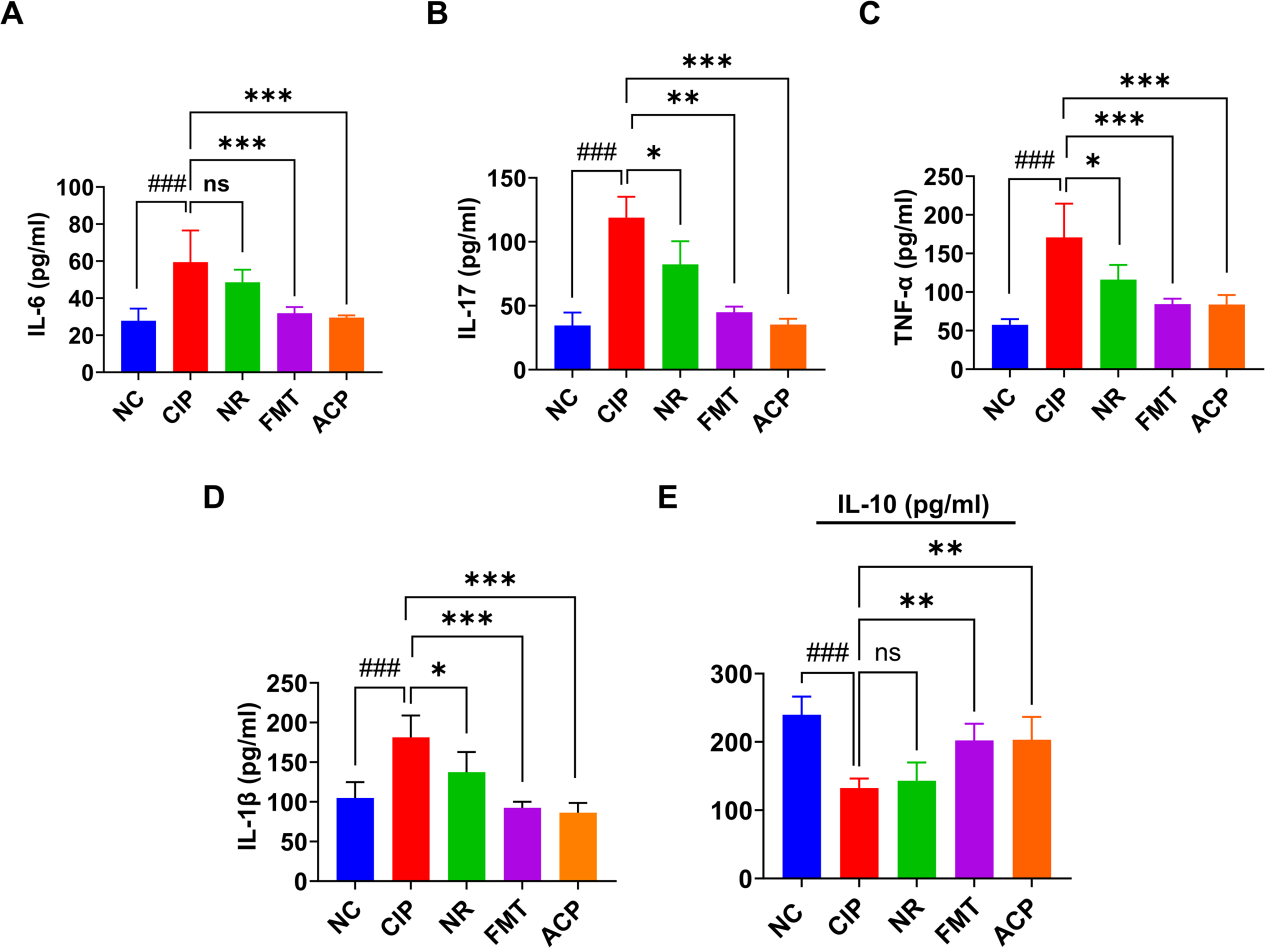
ACP and ACP-FMT modulate serum inflammatory cytokine levels. The level of pro-inflammatory cytokines in serum after antibiotic-induced inflammation is influenced by ACP and ACP-FMT groups. **(A)** IL-6, **(B)** IL-17, **(C)** TNF-α, **(D)** IL-1β, and anti-inflammatory cytokines **(E)** IL-10. Results are outlined as follows: mean ± SD, ns (not significant), *p < 0.05, **p < 0.01, ***p < 0.001, ACP and ACP-FMT vs. CIP group; ^###^*p* < 0.001 CIP vs. normal control group.

Serum concentrations of IL-6, IL-17, TNF-α, IL-1β, and IL-10 were measured using ELISA across all five groups. The CIP group showed significantly elevated levels of IL-6 (59.45 ± 17.13), IL-17 (119.0 ± 16.25), TNF-α (170.7 ± 43.84), IL-1β (181.3 ± 27.66), and IL-10 (132.4 ± 14.09) (*p* < 0.001 for all) compared to the normal control group. The NR group demonstrated partial reduction in cytokine levels but remained significantly elevated compared to the normal control group (IL-6: 48.66 ± 6.69; n) and (IL-17: 82.34 ± 18.15; *p* < 0.05), (TNF-α: 116.1 ± 18.93; *p* < 0.05), and IL-1β: (137.5 ± 25.29; *p* < 0.05). Both ACP and ACP-FMT treatment groups showed significant reductions in all four pro-inflammatory cytokines compared to the CIP and NR groups. Both ACP and FMT decreased IL-6 (ACP: 29.56 ± 1.10, FMT: 31.85 ± 3.39; *p* < 0.001 for both), TNF-α (ACP: 83.84 ± 12.39, FMT: 84.18 ± 7.25; *p* < 0.001 for both), and IL-1β (ACP: 86.42 ± 12.18, FMT: 92.42 ± 7.70; *p* < 0.001 for both), while ACP showed stronger downregulation of IL-17 (ACP: 35.31 ± 4.53; *p* < 0.001, FMT: 44.97 ± 4.39; *p* < 0.01), with levels approaching those of normal control. For IL-10, ACP, and FMT, notably improved the anti-inflammatory level (ACP: 203.1 ± 33.47, FMT: 202.0 ± 24.64; *p* < 0.01; for both). This highlights the superior anti-inflammatory efficacy of therapeutic interventions over NR alone, as shown in **Figure 6**.

### 3.7 ACP and FMT treatment restores bacterial taxonomic diversity in antibiotic-treated mice

**Figure 7:**
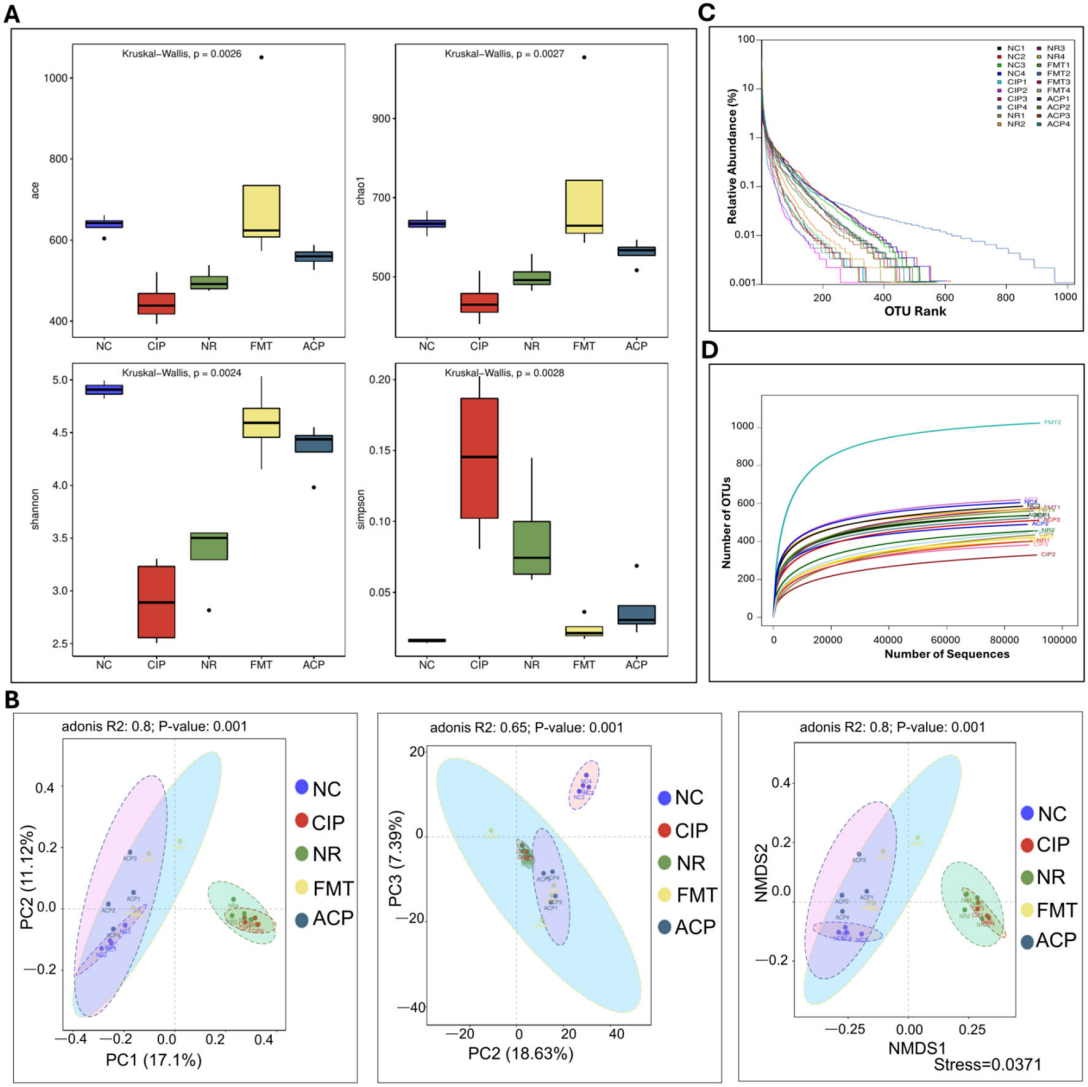
ACP and ACP-FMT restore gut microbial Alpha and Beta diversity. **(A)** Alpha diversity indices, Ace, Chao-1, Shannon, and Simpson assess richness, abundance, and diversity. Alpha diversity parameters depicting microbial richness and diversity across treatment groups. **(B)** Analysis of Beta diversity in microbial communities. Multidimensional Non-metric Scaling (NMDS) plots show beta-diversity patterns in mice treated with ACP, ACP-FMT, and CIP, highlighting how treatment categories are grouped in relation to the normal control group. Principal Component Analysis (PCA) three-dimensional structures that illustrate how treatment groups are arranged in relation to the normal control group and explain beta diversity patterns in mice treated with antibiotics and ACP. **(C)** Rank-abundance curves illustrate the relative abundance of microbial taxa across experimental groups, and **(D)** rarefaction curves provide insight into the microbial diversity of the samples.

16S rRNA sequencing was used to inspect gut microbiota structure and characteristics across all experimental groups, examining the impact of CIP-induced dysbiosis and comparing NR versus therapeutic interventions. To evaluate bacterial diversity and richness in all groups, alpha diversity parameters were analyzed, as illustrated in **Figure 7A**. Beta diversity analysis via principal component analysis (PCA) and non-metric multidimensional scaling (NMDS) explored microbial community composition across all groups. The CIP group clustered distinctly away from the NC group, showing severe dysbiosis. The NR group exhibited partial movement toward NC but remained notably separated. In contrast, ACP and ACP-FMT treatment groups clustered much closer to the normal control group, indicating much compositional similarity to healthy microbiota than NR alone. This revealed the superior efficacy of therapeutic interventions in restoring normal microbial community structure, as shown in **Figure 7B**. Rank abundance curves showed that the normal control group displayed high species richness with elongated curves. The CIP group presented dramatically reduced richness. The NR group demonstrated partial restoration but remained significantly lower than the normal control. Both ACP and ACP-FMT treatment groups indicated higher richness than the NR and CIP groups, with diversity indices approaching normal control levels, suggesting superior prebiotic effects of the therapeutic interventions, as displayed in **Figure 7C**. Rarefaction curve analysis confirmed comprehensive microbial data collection across all groups. Shannon diversity index and observed species counts revealed the following hierarchy: NC > ACP > ACP-FMT > NR > CIP. These findings confirm that, while NR partially restores microbial diversity, therapeutic interventions, ACP, and ACP-FMT more effectively restore species richness and evenness (**Figure 7D**).

**Figure 8:**
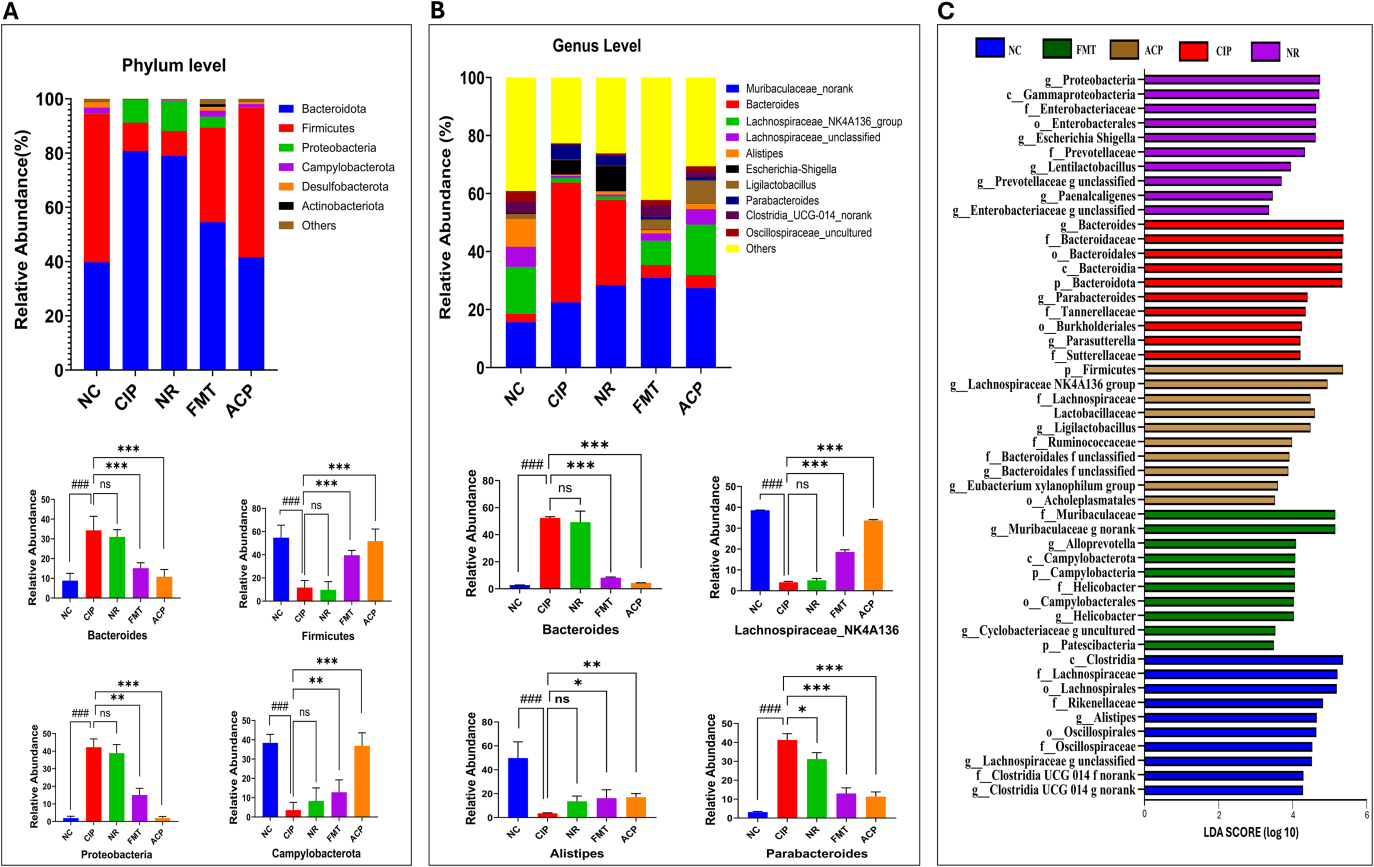
Modulation of gut microbiota ecology by ACP and ACP-FMT: Microbial diversity at different taxonomic levels: **(A)** Phylum, **(B)** Genus. ACP and ACP-FMT treatment restore microbial balance, enhancing beneficial taxa and mitigating antibiotic-induced dysbiosis. **(C)** LEfSe (v1.0) was conducted using an LDA score threshold of ≥ 2.0 and a significance level of *p* < 0.05. Taxa are grouped by treatment experimental groups, with the length of the bars representing the magnitude of the LDA (log10) score and the color indicating the treatment group in which the taxon is most abundant. Results are outlined as follows: mean ± SD, ns (not significant), *p < 0.05, **p < 0.01, ***p < 0.001, ACP and ACP-FMT vs. CIP group; ^###^*p* < 0.001 CIP vs. NC group.

Bacterial community proportional distribution was analyzed across all five groups following the 14-day CIP treatment and subsequent 14-day intervention period. Changes in bacterial abundance were examined at multiple taxonomic levels: phylum, order, family, genus, and species. At the phylum level (**Figure 8A**), the CIP group was dominated by *Proteobacteria* (42.25 ± 4.73; *p* < 0.001) with concomitant increase in *Bacteroidetes* (34.31 ± 7.10; *p* < 0.001), with dramatic reductions in *Firmicutes* (11.64 ± 6.07; *p* < 0.001) and *Campylobacterota* (3.56 ± 4.00; *p* < 0.001), compared to normal control. The NR group showed partial restoration (*Proteobacteria*: 36.86 ± 4.95; ns), (*Firmicutes*: 9.77 ± 7.04; ns), (*Campylobacterota*: 8.29 ± 6.76; ns), and (*Bacteroidetes*: 31.00 ± 3.63; ns), but significant imbalances persisted. Both ACP and ACP-FMT treatments nearly restored bacterial composition to baseline levels, with recovery of *Firmicutes* (ACP: 51.74 ± 10.44, FMT: 39.64 ± 4.11 *p* < 0.001; for both), and *Campylobacterota* (ACP: 36.91 ± 6.69; *p* < 0.001, FMT: 12.83 ± 6.38; *p* < 0.01), and reduction of *Proteobacteria* (ACP: 1.96 ± 0.87; *p* < 0.001, FMT: 15.07 ± 3.80; *p* < 0.01), and Bacteroidetes (ACP: 10.83 ± 3.67, FMT: 15.10 ± 2.82 *p* < 0.001; for both), effects that were superior to NR treatment alone. The class level, ACP and FMT were dominated by *Bacilli* and whereas the CIP and NR groups showed an increase in *Gammaprotobacteria* represented in **Supplementary Figure 1A**. Furthermore, we examined the order level. The CIP group showed predominance of *Enterobacteriales* and *Bacteroidales* with significant declines in *Oscillospirales* and *Lachnospirales*. The NR group demonstrated incomplete restoration of these orders. ACP and ACP-FMT treatments effectively rectified this imbalance, significantly increasing *Lachnospirales* and *Oscillospirales* richness compared to CIP and NR groups (**Supplementary Figure 1B**). Family-level analysis revealed that the CIP group showed substantial increases in potentially pathogenic families (*Bacteroidaceae*, *Tannerellaceae*, and *Enterobacteriaceae*) with significant decreases in beneficial families (*Lactobacillaceae*, Rikenellaceae, and Lachnospiraceae). The NR group showed partial but incomplete restoration. Both ACP and ACP-FMT treatments effectively restored these family-level alterations, showing superior efficacy compared to NR (**Supplementary Figure 1C**). At the genus level, the CIP group showed increased pathogenic genera and was dominated by *Bacteroides* (52.40 ± 0.81; *p* < 0.001) with dramatic reductions in beneficial genera, *Lachnospiraceae*-NK4A136 group (4.08 ± 0.43; *p* < 0.001) and Alistipes (3.48 ± 0.45; *p* < 0.001), and increased *Parabacteroides* (41.31 ± 3.35; *p* < 0.001), compared to normal control. Both ACP and FMT treatments nearly restored bacterial composition to baseline levels, with recovery of *Lachnospiraceae*-NK4A136 (ACP: 33.71 ± 0.40, FMT: 18.64 ± 1.03 *p* < 0.001; for both), and *Alistipes* (ACP: 16.99 ± 3.06; *p* < 0.01, FMT: 16.17 ± 7.04; *p* < 0.05), and reduction of *Bacteroides* (ACP: 4.38 ± 0.08; *p* < 0.001, FMT: 8.14 ± 0.57; *p* < 0.001), and *Parabacteroides* (ACP: 11.34 ± 2.45, FMT: 13.00 ± 2.98 *p* < 0.001; for both), effects that were superior to NR treatment alone. The NR group demonstrated incomplete genus-level restoration. ACP and ACP-FMT treatments recovered these imbalances to near-normal levels, as shown in **Figure 8B**. LEfSe analysis revealed distinct taxonomic signatures across experimental groups, reflecting differential microbiome compositions in response to treatment. The NC group was characterized by a predominance of members belonging to the *Lachnospiraceae* gut group, a family broadly associated with a healthy and balanced gut ecosystem. In contrast, the CIP group exhibited significant enrichment of *Bacteroides* species, a compositional shift widely recognized as an indicator of gut dysbiosis and microbiome destabilization following antibiotic exposure. The ACP-FMT group showed an elevated relative abundance of potentially beneficial taxa, notably *Muribaculaceae* and *Alloprevotella*, both of which have been associated with gut homeostasis and mucosal health. ACP-treated group was distinguished by an enrichment of *Firmicutes* members, particularly *Ligilactobacillus* species, which are identified for their probiotic properties and contributions to intestinal barrier function (**Figure 8C**). These findings demonstrated that both ACP and ACP-FMT treatments effectively change CIP-induced gut dysbiosis by restoring alpha and beta diversity indices toward levels more closely resembling those of the healthy control, while concurrently promoting the proliferation of health-associated bacterial taxa. These results collectively support the therapeutic potential of ACP and ACP-FMT as viable microbiome-targeted strategies for mitigating the adverse consequences of antibiotic-induced dysbiosis via targeted modulation of gut microbial community structure and function.

## 4 Discussion

Mushrooms are praised for their nutritional value and have been used as food and medicine since ancient times (42, 43). Nowadays, mushrooms are recognized as nutrient-dense, incorporated into functional foods, and used as meat substitutes (44). Mushrooms are a good source of protein, carbohydrates, and dietary fiber. They contain essential amino acids, various carbohydrates (sucrose, xylose, rhamnose, mannose, and fructose), and fatty acids. Mushrooms possess antioxidant and anti-inflammatory properties, which help in preventing and treating chronic diseases (45). *Agrocybe cylindracea* (*A. cylindracea*), known as the poplar mushroom, is an edible mushroom with increasing interest due to its nutritional value, potential medicinal benefits, and ease of cultivation (46). Studies have shown that *A. cylindracea* contains essential amino acids, B vitamins, vitamin D, potassium, phosphorus, calcium, and magnesium (47). Extracts from *A. cylindracea* exhibit antitumor and antioxidant activities (48).

In this study, we used polysaccharides from *A. cylindracea* (ACP) by the hot water extraction method. Polysaccharides from edible mushroom, *A. cylindracea*, are known for their diverse monosaccharide compositions, which influence their biological activities (49). These polysaccharides are considered bioactive macromolecules with potential prebiotic properties, influencing gut microbiota composition (33, 50).

Edible mushroom polysaccharides often contain glucose, galactose, mannose, xylose, and fucose as major components. The presence of glucuronic acid and galacturonic acid is also common, contributing to the acidic nature of some polysaccharides (51). The high glucose content is a common feature, as glucose often forms the backbone of fungal polysaccharides (52). Polysaccharides found in mushrooms, particularly β-glucans, have immunostimulant properties that are beneficial for modern medicine. Mushrooms are linked to antimicrobial, anti-diabetic, anticoagulant, and anticancer properties. They also have cholesterol-lowering effects. Ergothioneine, an amino acid found in mushrooms, may offer benefits for cardiometabolic health (53–55).

The frequent selection of BALB/c mice for dysbiosis experiments is due to their well-characterized immunological profile, particularly their Th2-biased immune responses, and their recognized use in gut microbiota research (56, 57). This extensive historical characterization provides a robust baseline for interpreting microbiome alterations, enabling more nuanced and comparable investigations into antibiotic-induced dysbiosis and host-microbe interactions (57). Overuse of antibiotics is a global public health challenge that accelerates antimicrobial resistance (58). Antibiotic-induced microbiota alterations can persist for weeks or even months post-treatment (59). CIP is a fluoroquinolone antibiotic used to treat a variety of bacterial infections (60). While effective against pathogens, CIP can significantly disrupt the gut microbiota, leading to dysbiosis (61). Antibiotics like CIP can diminish the diversity of gut microbiota, which is crucial for maintaining a healthy gut ecosystem. A decrease in diversity can impair various functions, including digestion, energy metabolism, and immune modulation (62, 63). High doses of CIP can impair gut barrier integrity and cause weight loss and anorexia (37). Histological examination of colonic tissue following antibiotic administration and subsequent polysaccharide treatment yielded consistent and corroborating results. Antibiotic exposure induced considerable mucosal damage, characterized by disrupted epithelial architecture, inflammatory cell infiltration, and a marked reduction in goblet cell density. Treatment with ACP, as well as ACP-FMT, effectively restored normal colonic histomorphology and significantly recovered goblet cell populations, indicative of mucosal regeneration and reinforced mucus barrier function. These findings collectively suggest a cytoprotective role of ACP against antibiotic-induced colonic injury, likely through modulation of mucosal integrity and immune homeostasis. These results align with previously reported therapeutic effects of *Pleurotus ostreatus* polysaccharides in mitigating antibiotic-associated intestinal damage, further supporting the broader premise that fungal-derived polysaccharides possess significant mucosal-restorative and gastroprotective potential (64, 65). FMT is being explored as a method to improve gut microbiota, and mushroom polysaccharides are being investigated for their potential to modulate gut microbiota (66, 67). The use of FMT with donor mice that have consumed mushroom polysaccharides aims to transplant the beneficial effects of these polysaccharides to a recipient organism (68). The mushroom polysaccharides and FMT could potentially enhance the therapeutic effects on gut microbiota. Supplementation with mushroom polysaccharides can create short-chain fatty acids (SCFA), increase the number of beneficial bacteria, and change the makeup and function of the gut microbiota (69, 70). In recipients with dysbiosis, transplanting this altered microbiota through FMT may aid in restoring gut balance.

Several illnesses have been connected to changes in the diversity and richness of the gut microbiota, which is essential for preserving general health (71). To assess antibiotic-induced gut dysbiosis, we used Illumina MiSeq platforms for 16S rRNA sequencing. with CIP and to evaluate ACP prebiotic potential and therapeutic impact. Alpha and beta diversity parameters were used to assess microbial diversity, richness, similarities, and differences between groups. Microbial diversity and richness decreased after 14 days of antibiotic administration, according to alpha diversity results. All these changes were reversed with improved alpha diversity after 14 days of ACP supplementation and ACP-FMT. The results demonstrated that samples from both the antibiotic-treated and natural recovery groups deviated from normal control. Notably, ACP-treated group and the ACP-FMT-treated group exhibited greater similarity to the normal control group, positioning them closer to the baseline values and indicating enhanced resemblance to normal conditions. The beta diversity analysis further confirmed distinct differences between the antibiotic-treated, natural recovery, and ACP treatment groups compared to the normal control, with ACP-FMT showing comparable patterns. These observations align with prior research investigating the therapeutic effects of polysaccharides derived from *Auricularia auricula* on antibiotic-associated diarrhea, where analogous outcomes were documented (72).

In this investigation, we examined bacterial abundance across multiple taxonomic levels, encompassing phylum, order, family, genus, and species classifications. Our findings demonstrated that antibiotic treatment substantially altered the gut microbiota composition compared to the normal control group, whereas ACP and ACP-FMT interventions showed restorative effects. At the phylum level, antibiotic exposure significantly modified bacterial community structure, characterized by an elevation in *Proteobacteria* abundance, a reduction in *Firmicutes*, and an increase in the relative proportions of *Bacteroidetes*. Notably, both ACP and ACP-FMT interventions facilitated the restoration of microbial equilibrium toward baseline levels. Order-level analysis revealed that *Enterobacteriales* and *Bacteroidales* were dominant in both antibiotic-treated and natural-recovery groups, with diminished representation of *Oscillospirales* and *Lachnospirales*. The administration of ACP and ACP-FMT enhanced the abundance of *Lachnospirales* and *Oscillospirales* orders. At the family level, antibiotic-induced modifications were characterized by elevated abundances of *Bacteroidaceae*, *Tannerellaceae*, and *Enterobacteriaceae*, alongside decreased levels of *Lactobacillaceae*, *Rikenellaceae*, and *Lachnospiraceae*. These disturbances were ameliorated following ACP and ACP-FMT treatment. Genus-level examination demonstrated increased abundances of *Bacteroides*, *Parabacteroides*, and *Escherichia-Shigella* in antibiotic-treated and natural recovery groups, whereas ACP and ACP-FMT treatment groups displayed recovery patterns approaching normal control values. At the species level, antibiotic administration resulted in elevated *Escherichia coli* abundance while diminishing *Lachnospiraceae*-NK4A136 and *Muribaculaceae* species. Both ACP and ACP-FMT treatments successfully counteracted these antibiotic-induced taxonomic shifts in the gut microbiota. These findings are concordant with previous investigations examining the therapeutic potential of polysaccharides from *Brassica rapa* L., Deglet Noor dates, and *Artocarpus heterophyllus* Lam. polysaccharides on intestinal damage and their modulatory effects on gut microbial communities (73–75).

Antibiotics may directly affect goblet cells, which are responsible for mucin production. This can lead to a reduction in the amount of Mucin-2 produced, weakening the mucus layer (76). With the dysbiosis of beneficial bacteria, there can be an overgrowth of pathogenic bacteria. These pathogens can degrade the mucus layer, further compromising the intestinal barrier (77). In the present study, immunohistochemical analysis was conducted to evaluate Mucin-2 expression levels in colonic tissue samples. The findings demonstrated that a 14-day CIP administration significantly reduced Mucin-2 expression compared to the control group. Notably, subsequent therapeutic intervention with ACP and ACP-FMT over 14 days substantially restored Mucin-2 expression in the colon tissue. These observations align with findings from prior research in the field, thereby corroborating our experimental outcomes (29).

Antibiotics can modulate the production of pro-inflammatory cytokines, impacting the host’s immune and inflammatory responses. This modulation is complex and depends on factors such as the type of antibiotic, the specific cytokines involved, and the overall context of the infection or inflammatory condition (78). Pro-inflammatory cytokines like TNF-α, IL-1β, and IL-6 are crucial for initiating and sustaining immune responses. However, their excessive production can lead to detrimental effects, including tissue damage and organ dysfunction (79, 80). Concurrently, our data demonstrated elevated abundance of *Bacteroides* and *Proteobacteria* at the phylum level, taxa commonly associated with enhanced production of pro-inflammatory cytokines. Administration of ACP and ACP-FMT significantly reduced cytokine expression levels following antibiotic-induced dysbiosis, indicating modulation of the intestinal microbiota and amelioration of the inflammatory response. These findings underscore the therapeutic potential of ACP and ACP-FMT in effectively mitigating antibiotic-induced inflammation and enhancing immune function.

Antibiotics can disrupt intestinal tight junction barriers, which are critical for intestinal homeostasis (81). Antibiotics, especially broad-spectrum ones, can significantly alter the composition and diversity of the intestinal microbiota. This dysbiosis can reduce the production of beneficial metabolites, such as SCFA, essential for maintaining gut barrier integrity. SCFA like butyrate are crucial for providing energy to colonocytes and enhancing tight junction protein expression. The reduction in SCFA due to dysbiosis can compromise tight junction function (82, 83). Immunofluorescence staining analysis revealed that antibiotic treatment significantly downregulated the expression of tight junction proteins, consistent with compromised epithelial barrier integrity. Conversely, ACP treatment markedly upregulated the expression of these proteins, effectively restoring gut barrier function toward levels comparable to those observed in the normal control group. These findings are aligned with previously published reports, further corroborating the barrier-protective properties of fungal-derived polysaccharides in the context of antibiotic-induced intestinal injury (84, 85), which demonstrated that polysaccharides enhance the expression of tight junction proteins and Mucin-2, thereby strengthening intestinal barrier integrity. While a direct functional permeability assay, such as serum LPS, was not performed, the consistent restoration of Mucin-2 expression and tight junction proteins observed in this study provides strong structural and molecular evidence of improved intestinal barrier integrity following ACP and ACP-FMT treatment. The present study offers valuable insights into the therapeutic efficacy of ACP and ACP-FMT in alleviating antibiotic-induced gut dysbiosis; however, certain limitations should be acknowledged, including the absence of fecal short-chain fatty acid quantification and a dedicated ACP-only control group. Future investigations incorporating targeted metabolomics, functional pathway validation, and systematic safety evaluations will further consolidate the therapeutic profile of ACP in antibiotic-associated dysbiosis.

## 5 Conclusion

This study demonstrates that ACP effectively restores gut microbiota diversity and intestinal homeostasis following CIP-induced dysbiosis by enhancing barrier integrity, promoting mucosal healing, and attenuating inflammatory responses. Strikingly, ACP-FMT treated donors successfully transplanted these therapeutic benefits to dysbiotic recipients, evidenced by the recovery of important beneficial taxa including *Lactobacillaceae*, *Rikenellaceae*, and *Lachnospiraceae*, conclusively confirming that ACP restorative effects are primarily microbiota-mediated. Collectively, these findings position ACP as a promising, standardized, and scalable prebiotic alternative to conventional FMT for managing antibiotic-associated dysbiosis, with meaningful potential for future clinical applications.

## Data Availability

The data presented in this study are deposited in the NCBI Sequence Read Archive (SRA) repository, BioProject accession number PRJNA1477999.
