# Supplementary material for: Agrocybe cylindracea polysaccharides and polysaccharides-conditioned fecal microbiota transplantation similarly restore ciprofloxacin-induced microbial dysbiosis and improve intestinal barrier function: a comparative study
Source: Front Immunol. 2026 Jul 13;17:1841989. doi: 10.3389/fimmu.2026.1841989 (PMC13402469; doi:10.3389/fimmu.2026.1841989)
Supplement: Supplementary file 1 [file Supplementaryfile1.docx]

***Agrocybe cylindracea* Polysaccharides and Polysaccharides-Conditioned Fecal Microbiota Transplantation Similarly Restore Ciprofloxacin-Induced Microbial Dysbiosis and Improve Intestinal Barrier Function: A Comparative Study**

Aamna Atta ^1^, Muhammad Naveed ^1^, Jinting Liu ^1^, Immad Ansari ^1^, Renzhen Ma ^1^, Xiyu Wang ^2^*, Bin Feng ^1^*

^1^ College of Basic Medical Science, Dalian Medical University, Dalian 116044, China.

^2^ Department of Traditional Chinese Medicine, Dalian University Affiliated Xinhua Hospital, Dalian 116000, China.

[aaminaatta999@gmail.com](mailto:aaminaatta999@gmail.com) (A.A.); [naveed.uop10@gmail.com](mailto:naveed.uop10@gmail.com) (M.N.); [liujt202205@163.com](mailto:liujt202205@163.com) (J.L.); [Immadansari@outlook.com](mailto:Immadansari@outlook.com) (I.A.) ; [mrz200010@163.com](mailto:mrz200010@163.com) (M.R);

* Correspondance :

Email: [binfeng@dmu.edu.cn](mailto:binfeng@dmu.edu.cn) (B.F.); [28032929@qq.com](mailto:28032929@qq.com) (X.W.)





**Figure S1.** Modulation of gut microbiota ecology by ACP and ACP-FMT: Microbial diversity at different taxonomic levels: (**A**) Class, (**B**) Order, and (**C**) Family. ACP and ACP-FMT treatments restore microbial balance, enhance beneficial taxa, and mitigate CIP-induced dysbiosis.

**Table S1: Histopathological score: colon tissues were examined for inflammation and regeneration.**

|  | **Score** | **Significance** |
| --- | --- | --- |
| **Regeneration** | 4 | Absent Regeneration |
|  | 3 | Epithelial Disruption |
|  | 2 | Regeneration with crypt depletion |
|  | 1 | Near complete regeneration |
|  | 0 | Normal tissue |
| **Inflammation** | 3 | High-grade injury |
|  | 2 | Intermediate Injury |
|  | 1 | Mild Injury |
|  | 0 | None |
